# Psychological Testing in a Complex Case: Psychosis in Co-occurring PTSD, Gender Dysphoria, and Bipolar Disorder

**DOI:** 10.7759/cureus.49626

**Published:** 2023-11-29

**Authors:** Maliha I Ansari, Susan D Sperry, Luba Leontieva, James L Megna

**Affiliations:** 1 Psychiatry and Behavioral Sciences, State University of New York Upstate Medical University, Syracuse, USA; 2 Psychology, State University of New York Upstate Medical University, Syracuse, USA; 3 Psychiatry, State University of New York Upstate Medical University, Syracuse, USA

**Keywords:** gender dysphoria, ptsd diagnosis and treatment, cannabis use disorder, schizoaffective disorder, bipolar disorder

## Abstract

This case report investigates the concurrent presence of post-traumatic stress disorder (PTSD) and bipolar disorder (BD) in the transgender population. We present a case involving a 21-year-old female-to-male transgender individual (preferred pronouns - they/them). The patient had a history of psychosis, trauma, gender dysphoria (GD), inconsistent hormone (testosterone) treatments, and a self-attributed diagnosis of "associative identity disorder" with 21 distinct "identities." They had two emergency admissions in quick succession, both characterized by analogous symptoms. Contributing factors included a recent discontinuation of antipsychotic medications and a history of cannabis use. Their family history included BD in the patient's mother and schizophrenia in their paternal grandfather. The differential diagnoses considered were brief psychosis, BD, PTSD, and substance-induced mania/psychosis. A notable improvement in the patient's clinical presentation was observed during their hospital stay. Their therapeutic regimen comprised olanzapine, hydroxyzine, topiramate, trazodone, and lithium carbonate extended-release. Additionally, the patient underwent psychological testing. This progress solidified the primary diagnosis as PTSD coexisting with BD, manifesting episodes of mania and psychosis. This report highlights the critical role of psychological evaluations in assessing symptoms in patients with multiple psychiatric co-morbidities. Our findings emphasize the importance of a comprehensive, multidisciplinary approach for accurate diagnosis and efficacious treatment of such intricate cases.

## Introduction

Post-traumatic stress disorder (PTSD) is a common and debilitating psychiatric condition that may develop after exposure to a traumatic event. With a lifetime prevalence of 6.1% [1], PTSD is characterized by intrusive thoughts related to the event, avoidance of reminders of the event, negative alterations in mood and cognition, and heightened arousal and reactivity [2]. Meanwhile, bipolar disorder (BD) is an impairing, progressive illness affecting up to one in 25 individuals [3]. The diagnosis encompasses a full range of chronic mood and maniform disorders with heterogeneous clinical presentations and longitudinal courses [4,5]. Patients with BD who have comorbid anxiety disorders [5,6] and a history of childhood maltreatment have the poorest clinical course characterized by early onset of disease, rapid cycling, and a greater number of manic and depressive episodes. In turn, patients with poor courses of illness are at increased risk of comorbid anxiety disorders, PTSD, and completed suicide (i.e., 4% of people with BD); hence, they are associated with negative clinical outcomes [5-8]. Being aware of how commonly PTSD and BD co-occur could inform strategies to improve diagnostic accuracy, treatment of both disorders’ symptoms, and support for psychosocial problems in an individual.

Transgender and gender-diverse (TGD) people experience violence and trauma exposure and subsequent PTSD and suicidality at higher rates than the general population [9]. TGD people are prominent targets of discrimination, which increases the risk for mental health struggles [10]. A study of over 25,000 TG individuals in inpatient psychiatric encounters found that 77% of encounters involving TGD individuals had at least one mental health diagnosis, depression, anxiety, and psychosis being the most common, relative to 37.8% of hospital encounters involving cisgender individuals [11].

This case study examines the comorbidity of PTSD and BD in the presentation of a transgender (TG) patient who was hospitalized secondary to psychotic decompensation. The patient had a history of trauma, gender dysphoria (GD), anabolic steroid use for medical transition, and a family history of BD. They initially presented with frank psychosis with mania. We highlight the epidemiology and importance of assessing bipolar and PTSD symptoms together, along with the importance of psychological testing in the diagnosis of such cases.

## Case presentation

The patient was a 21-year-old female-to-male TG individual (preferred pronouns - they/them), domiciled in the US, living with their father and younger brother, presented to our hospital's Emergency Department (ED) with signs of thought disorder, including disordered speech, thinking, and behavior, as well as prominent persecutory delusions. The patient had a personal history of attention deficit hyperactivity disorder (ADHD), trauma, and GD and had, in the past year, begun hormonal treatment to transition to male; they also had a family history of both BD and schizophrenia. Over the previous eight months, the patient, per the family report, had increased emotional lability and became markedly disorganized in thoughts and behaviors. At admission, they had difficulty sleeping and had increased goal-directed behaviors, such as frequent cleaning. The patient’s speech was rapid, pressured, tangential, loosely associated, and disorganized, and they were distractible but fixated on persecutory delusional ideation. They reported that they were afraid of white men, their mother, and hospital staff due to past trauma. They reported a trauma history from being assaulted and abused in hospitals and experienced paranoia with worsening fears at night. They reported illusions, such as seeing a chair and seeing it as something ominous, and significant loose associations, which led them to state that they had a more severe form of dissociative identity disorder (DID), which they called “associative identity disorder.” They also maintained that they had multiple personalities, stating that they had 21 “alters” since they were 21 years old.

Seven days prior to their current admission, the patient had been discharged from inpatient psychiatric care after being hospitalized for seven days. At presentation for the first admission, they also evidenced significant thought disorganization, persecutory ideation, and pressured speech and were noted to speak in multiple voices and refer to themself as “we.” Among their reported identities were "Zen," a "persecutor" who was "black," and "Anna,” who was a six-year-old girl who stated, "XX [patient’s name] needs help." At that admission, they spoke perseveratively about an alleged sexual assault by a psychiatric provider at another hospital whom they asserted was a sex trafficker on the FBI’s Most Wanted List. Just prior to that previous hospitalization, the patient began to assert that they had multiple identities and had self-reported amnesia, stating that, “We have forgotten but we are trying to remember,” and “We don’t want to remember.” In the unit, the patient frequently changed pronouns, and occasionally spoke in the third person; they used the pronoun “we” while referring to themselves and/or their identities, and used “I” while speaking to others as one of the identities. The patient had identified as TG, had begun hormone treatments to medically transition, and had wanted to legally change their name to their 19-year-old boy identity’s name before their psychosis became evident. After discharge, they continued to have worsening psychotic symptoms despite being medication adherent.

The patient’s father reported that the patient had experienced greater mood disturbance, delusions, thought disorganization, and erratic behavior possibly since they discontinued their quetiapine eight months ago. They had also been using hormones for gender transition somewhat “erratically,” with the father being uncertain of the timing of treatment. The father noted delusions, disorganization, and increased insomnia over the past week, as well as their recent endorsement of multiple identities. Previously, these issues were present but milder in severity and more intermittent. The patient’s delusions revolved around their interactions with the medical staff at another hospital where they accused the staff members of sexually assaulting them even though, per the father, the patient had never been alone with the doctors. The father expressed concerns about the patient’s safety, as they had made statements about killing themselves and their mother.

The differential diagnoses for this patient included brief psychosis possibly due to an unknown stressor, BD, PTSD, and substance-induced mania/psychosis (cannabis). Clinically relevant information included their historical diagnoses of depression, anxiety, and borderline personality disorder, their prior suicide attempts, and their initial presentation with delusional thinking and dissociative symptoms. Throughout the patient's stay, there were concerns for a primary psychotic (possibly manic) process, given their initial presentation and the depth and breadth of delusional thinking. There were also concerns about interpersonal and coping deficits and trauma-related dissociative symptoms. Substance-induced mood or psychotic symptoms were less likely, as the patient had been abstinent from the substances during their admission. The patient’s behavior and thought processes gradually improved over the course of their stay, with improved sleep, speech, and engagement in conversations without getting distracted. However, delusional thinking and fixed non-redirectable delusions were still present, and they continued to endorse multiple identities upon being discharged. The improvement in the patient’s behavior and thought processes over the course of their stay, along with concerns about trauma, suggested a possible diagnosis of PTSD and BD with current episodes of mania and psychosis.

Family history

Per a collateral report from the patient’s father, the patient’s mother had been diagnosed with BD, and they also had a family history of schizophrenia in their maternal uncle and maternal grandfather.

Developmental history and early care

The patient’s mother was not actively involved in the patient’s care as a young child, often neglecting the patient’s needs. The mother-child relationship suffered and had several difficulties, as reported by the father, who underwent a divorce during the patient’s adolescence and subsequently became their sole parent. The patient regarded their mother as someone who had traumatized and emotionally abused them and left them with fears of rejection and abandonment. Instead, the patient’s support reportedly came from their father, aunt, and paternal grandmother. They stated that they were closest to their father, but had to depend solely on their mother for support after their father was diagnosed with chronic regional pain syndrome (CRPS) and bedridden for three years during the patient’s early teenage years.

In school, the patient faced organizational skill problems from grades 1-3, which were labeled as “attention-seeking behaviors” by the school authorities. The patient’s need for help was reportedly often dismissed by the teachers in school whom they looked up to. They attended a normal public school with an Individual Education Plan (IEP) but dropped out during the eighth grade due to being the recipient of bullying and sexual assault (per collateral from the father). The patient was reportedly diagnosed with ADHD by their primary care provider (PCP) and autism spectrum disorder (ASD) as a teenager.

The patient’s presentation did not suggest neurologic illness or cognitive impairment; therefore, neuropsychological testing was deemed unwarranted. Testing of overall cognitive function (IQ) was not completed as the patient’s psychiatric presentation did not appear to be complicated by their overall cognitive function, and they was fully able to engage in services on the unit, including therapy, groups, and assessments.

Previous medications and drug use

The patient had been prescribed quetiapine, 100 mg BID, by an outpatient psychiatric clinic when the patient was 13 years old. This was their first psychiatric encounter as per the chart review and collateral information (Table 1). The patient had one prior psychiatry ED visit where they had been on a 72-hour hold. The patient weaned themselves off of quetiapine eight months prior to their first hospitalization at our hospital. They had also stopped taking topiramate two days before their current presentation to the ED and had reportedly not slept since. The patient started testosterone therapy again three weeks ago after a period of erratic therapy due to insurance issues. Additionally, they had reportedly been micro-dosing cannabis over the past three years. At their first admission, their urine toxicity screening was positive for cannabinoids and benzodiazepines; at the second admission, it was positive for cannabinoids.

**Table 1:** Psychiatric encounter timeline of the patient CPEP: Comprehensive Psychiatric Emergency Program, PRN: as needed

Psychiatric encounter	Timeline	Patient Presentation	Medications
Outpatient psychiatric clinic	2013	Auditory hallucinations, anxiety	Quetiapine 100 mg BID
Psychiatry hospital emergency hold for 72 hours (CPEP)	03/2023	Thought content abnormal, developed delusions regarding being assaulted by a psychiatric provider at this hospital	PRN IM antipsychotics
Inpatient psychiatric hospitalization (our facility)	03/2023	Thought disorganization, persecutory ideation, and pressured speech, spoke in multiple voices, referring to themself as “we”	Olanzapine, hydroxyzine, topiramate
Current inpatient Psychiatric hospitalization (our facility)	04/2023	Disordered speech, thinking, behavior, and, persecutory delusions	Olanzapine, hydroxyzine, trazodone, lithium carbonate extended release

Pharmacologic interventions

At their initial admission to inpatient psychiatric care, the patient was started on olanzapine and hydroxyzine. On the third day, topiramate was added to manage bipolar symptoms. At their second admission, olanzapine, hydroxyzine, and topiramate were restarted, and trazodone was added for sleep. On the third day, topiramate was discontinued, and lithium carbonate extended-release was added. There were considerable changes in the patient’s behavior and cognition while on lithium (e.g., a more organized, linear, and coherent thought process); however, their thought content remained delusional. The patient stated that their “DID was gone” and that now “[they] had C-PTSD” or “Complex-PTSD.”

Psychological findings

After 13 days, the patient underwent a psychological assessment for differential diagnosis, which included a clinical interview, the Minnesota Multiphasic Personality Inventory - Third Edition (MMPI-3), and the Thematic Apperception Test (TAT) - a projective test in which the patient is asked to generate stories for several images, thereby providing insight to the patient’s thought processes and content and their views of themselves, others, and the world.

On the MMPI-3, the patient’s pattern of responses suggested some overreporting of psychopathology and somatic symptoms, which reflected intense psychological distress and their disclosed history of medical problems. The highest clinically significant elevation was on a scale reflecting dysfunctional negative emotions (RC7, T = 82), suggesting intrusive ideation, excessive worry, sleep difficulties, stress reactivity, and negative emotions, including re-experiencing traumatic events. Their second-highest clinically significant elevation was related to somatic complaints and preoccupation with their health (RC1, T = 80), which was consistent with the patient’s medical history. Their profile also contained an elevation on a hypomanic activation scale (RC9, T = 72), suggesting mood instability, euphoria and excitability, restlessness and boredom, and activation that manifested as impulsivity and behavioral disinhibition. Their profile also showed elevations on two personality scales, psychoticism (T = 71) and negative emotionality (T = 68). The elevation of psychoticism suggested that the patient experienced unusual thought processes and perceptual phenomena and was alienated from others, engaged in unrealistic thinking, and presented with impaired reality testing. The elevation on negative emotionality/neuroticism paralleled the elevation on RC7 and suggested they experienced various negative emotions, including anxiety, insecurity, and worry and that they were inhibited behaviorally, guilt-prone, and self-critical.

Responses to five cards from the TAT were disorganized and impoverished, with bizarre content. Their stories featured characters who were caring and empathic, and who felt invalidated, unheard by others, alienated, tired, and weary. Recurring themes included optimistic futures, loss, chaotic mental states, and pregnancy and childbirth. Characters provided and needed assistance and overcame personal and interpersonal conflicts in order to collaborate or achieve happiness. Although the patient described positive endings, their descriptions were often vague and impoverished. For example, in the story in which the man did not listen to the woman’s advice, the patient ended the story by stating that the characters all “got together” and “worked towards an option that would work best for all of them,” but did not provide any details about how this solution was achieved. When asked to infer characters’ emotional states, they occasionally responded with somatic complaints (e.g., feeling tired) in lieu of emotions or provided mildly valenced emotions, such as a character feeling “a bit sad,” “melancholy”, or “upset.” The content of the patient’s stories was often odd or unusual, especially in response to unstructured stimuli (e.g., the blank card). When asked what was happening at the moment, they responded, “Being clear as day…a clear sky.” In this story, they reported that the characters involved were “me, myself, and I,” with each experiencing discrete and unaligned feelings, potentially reflecting the multiple identities that the patient described in the clinical interview. In addition, they referenced faint lines that were visible from the shadows and creases of the paper and described characters who experienced “non-linear thought patterns.” They also perseverated on the concrete whiteness of the blank image, with vague, abstract, and unusual responses (e.g., “[They] can see everything in nothing and nothing in everything because white is the color of everything but also nothing, and so is black, so black is the color of everything and nothing as well.”).

The patient’s reports during the clinical interview, their MMPI-3 profile, and records associated with their current and prior admissions suggested clinically significant psychological distress associated with multiple traumatic events involving physical abuse. The patient’s distress was characterized by intrusive ideations, re-experiencing of these events, distressing negative emotions, and negative alterations in their mood and cognitions, including paranoia, which suggested a diagnosis of PTSD. Of note, the patient’s trauma experiences likely influenced the nature and expression of their psychotic symptoms (i.e., persecutory and paranoid delusions). The patient also evidenced tangentiality, loose associations, derailment, flight of ideas, significant disorganization/illogicality in their speech and thought process, decreased need for sleep, increased talkativeness, racing thoughts, psychomotor agitation, and a tendency to engage in risky, impulsive behaviors, which strongly suggested a diagnosis of BD, type I with mood-congruent psychotic features. This diagnosis was further supported by an elevation on the Hypomania scale in their MMPI-3 profile, as well as their family history of Bipolar I diagnoses. A primary psychotic disorder such as schizophrenia or schizoaffective disorder was not supported due to a lack of elevations on MMPI-3 scales reflecting aberrant experiences (RC8, T = 63), ideas of persecution (RC6, T = 55), and thought dysfunction (THD, T = 63). However, the patient demonstrated several features of schizotypal personality disorder during the clinical interview, on the MMPI-3 (psychoticism, T = 71) and on the TAT; these features included magical thinking, unusual perceptual experiences, odd cognitions and speech, suspiciousness and paranoid ideation with self-referential thinking, and an unusual understanding of themself. However, because the patient presented to the assessment during an acute manic episode associated with BD, type I, it remained unclear whether BD versus another disorder, such as schizotypal personality disorder, best explained their features of psychoticism.

## Discussion

The complexity of our case lies in the diagnostic difficulties of a patient presenting to our hospital for the first time with mania, psychosis, comorbid PTSD, and GD.

PTSD prevalence among individuals with BD is higher than the general population’s lifetime prevalence of PTSD of 6.80, with a higher prevalence in women with BD compared to men [12]. The estimated prevalence of BD in people with PTSD is 6%-55% higher than the estimated BD prevalence within the general population of 4.40 [13]. The elevated prevalence of PTSD among those with BD reflects the high rate of exposure to traumatic events in those with BD. Among individuals with BD, prior history of trauma, especially childhood trauma [14], occurred about twice as often as those with a diagnosis of PTSD in adulthood. About half of adults with BD experienced childhood abuse, which was significantly associated with longer reported duration of untreated BD, and greater severity of depressive and manic symptoms. This is consistent with the personal history of our patient, who reportedly suffered childhood abuse and neglect. Furthermore, concurrent PTSD and prior trauma exposure were both associated with a worse course of adulthood BD and more severe psychosocial impairment [15]. Our patient presented with significant persecutory delusions and paranoia that were likely related to their historical trauma. They also reported severe psychosocial impairment (e.g., difficulties interacting with certain men and an inability to leave their house to do chores), which improved upon treatment with mood stabilizers. A history of childhood maltreatment may be used as an indicator of disease progression to identify patients with BD who are at high risk for unfavorable clinical features and worse course of illness. This contribution to risk stratification could be used to improve the delivery of effective interventions. 

Our patient also suffered from GD. Meyer’s minority stress theory suggests that sexual minorities experience stress due to stigma, internalized homophobia, and hostility. It has been well-documented that gender minorities are more likely to have been victims of childhood sexual abuse [16-19]. Consequently, this lifetime of maltreatment and victimization negatively impacts mental and physical health outcomes [20]. Although less researched, the co-occurrence of GD with PTSD has been reported. In a study of 319,430 patients diagnosed with mood disorders and suicidal behaviors, which was further sub-grouped by co-diagnoses of GD in 4,840 patients, the prevalence of PTSD was found to be 22.5% in all patients with mood disorders, and 28.2% in the cohort with comorbid mood disorders and GD [21,22]. Trauma-related psychological conflicts may have contributed to our patient’s desire to change their gender from female to male.

The patient had been using cannabinoids to improve their mood symptoms and had self-weaned themselves off of antipsychotics during a period of erratic testosterone injections in higher than average doses, which raises the question of whether these factors contributed to the patient’s manic episode with psychotic symptoms. However, the presence of ongoing psychotic and manic symptoms during a period of sustained sobriety without testosterone suggested that these psychiatric symptoms were not solely attributable to substance and hormone use.

Psychological testing can provide valuable insight when a psychological diagnosis is uncertain. It can help rule out incorrect diagnoses that might have otherwise been given to the patient, which might have in turn led to incorrect treatment measures that worsened the patient’s condition. Our patient was “misdiagnosed” with ASD in the past and this diagnosis could not be verified through prior provider records. In the literature, the co-occurrence of ASD with GD has been higher than in the general population [23]. Further, on presentation, the patient self-reported DID, along with a history of childhood trauma, which many patients with DID are known to have [23]. Upon completing the psychological assessment, the patient did not meet the diagnostic criteria for ASD or DID; instead, their dissociative experiences seemed related to trauma history and identity issues secondary to GD. Although Soldati et al. suggest that GD patients with memory lapses and dissociative symptoms should be assessed for DID, diagnostic tests such as the DES, DDIS, and the SCID-D-R9 may be helpful, as recommended by the International Society for the Study of Trauma and Dissociation (ISSTD) [21]. The DES was administered to the patient but was considered invalid, as the patient gave a positive response to almost all of the items. Hence, in cases such as these, it becomes important to understand the underlying diagnosis - as literature suggests - to withhold hormonal therapy and/or gender-affirming surgery until the DID is treated. In our case, after treatment with lithium, the patient decided against pursuing hormone treatment for their GD and instead chose to prioritize their mental health care. This case highlights the importance of not blindly following previous diagnoses as this can lead to further miscalculations on the part of the physician and subsequently delay vital care.

## Conclusions

Our case underscores the diagnostic challenges involved in assessing individuals with multiple intersecting mental health conditions. The co-occurrence of BD, PTSD, and GD in our patients exemplifies the intricate interplay between traumatic experiences, psychological distress, and gender identity struggles. The significance of using psychological testing to clarify uncertain diagnoses and guide appropriate treatment cannot be understated. Our case further demonstrates how childhood trauma can cause and/or worsen mental health outcomes in individuals’ adulthoods. We also learned how a person’s decision to transition genders can be influenced by various factors, and a thorough screening should be done before hastily starting or withholding gender-conforming treatment. Overall, this case emphasizes the need for a multidimensional approach to mental health assessments that considers the interconnections between trauma, psychiatric disorders, and gender identity. By recognizing and addressing these complex dynamics, healthcare providers can ensure more accurate diagnoses, improve treatment outcomes, and deliver effective interventions tailored to the unique needs of each individual.
